# Large Scale Triboelectric Nanogenerator and Self-Powered Pressure Sensor Array Using Low Cost Roll-to-Roll UV Embossing

**DOI:** 10.1038/srep22253

**Published:** 2016-02-24

**Authors:** Lokesh Dhakar, Sudeep Gudla, Xuechuan Shan, Zhiping Wang, Francis Eng Hock Tay, Chun-Huat Heng, Chengkuo Lee

**Affiliations:** 1 Department of Electrical and Computer Engineering, National University of Singapore, 4 Engineering Drive 3, Singapore 117576; 2 NUS Graduate School for Integrative Sciences and Engineering, Centre for Life Sciences (CeLS), 28 Medical Drive, Singapore 117456; 3 Singapore Institute of Manufacturing Technology (SIMTech), 71 Nanyang Drive, Singapore 638075; 4 Department of Mechanical Engineering, National University of Singapore, 9 Engineering Drive 1, Singapore 117575.

## Abstract

Triboelectric nanogenerators (TENGs) have emerged as a potential solution for mechanical energy harvesting over conventional mechanisms such as piezoelectric and electromagnetic, due to easy fabrication, high efficiency and wider choice of materials. Traditional fabrication techniques used to realize TENGs involve plasma etching, soft lithography and nanoparticle deposition for higher performance. But lack of truly scalable fabrication processes still remains a critical challenge and bottleneck in the path of bringing TENGs to commercial production. In this paper, we demonstrate fabrication of large scale triboelectric nanogenerator (LS-TENG) using roll-to-roll ultraviolet embossing to pattern polyethylene terephthalate sheets. These LS-TENGs can be used to harvest energy from human motion and vehicle motion from embedded devices in floors and roads, respectively. LS-TENG generated a power density of 62.5 mW m^−2^. Using roll-to-roll processing technique, we also demonstrate a large scale triboelectric pressure sensor array with pressure detection sensitivity of 1.33 V kPa^−1^. The large scale pressure sensor array has applications in self-powered motion tracking, posture monitoring and electronic skin applications. This work demonstrates scalable fabrication of TENGs and self-powered pressure sensor arrays, which will lead to extremely low cost and bring them closer to commercial production.

Our daily lives are getting increasingly surrounded and connected by electronic devices and sensors in pursuit of improving the quality of human life. Majority of these sensors and devices run on batteries, which need to be charged and replaced at regular intervals. Harvesting irregular ambient mechanical energy^1^,^2^ in various forms e.g. human motion^3^, wind^4^,^5^, vibrations^6^ etc. has been proposed as one of the approaches to charge these batteries to reduce the need for charging on a regular basis^7^. Traditionally, piezoelectric^8^,^9^,^10^,^11^, electromagnetic^12^ and electrostatic mechanism^13^,^14^,^15^ based devices have been used to convert mechanical energy into usable form of electrical energy. Recently, triboelectric nanogenerators (TENG) have emerged as a potential solution to harvest mechanical energy available in the surroundings^16^,^17^,^18^,^19^. TENGs have advantage of easy fabrication, high efficiency and wider choice of materials over other traditional approaches such as piezoelectric and electromagnetic devices^20^,^21^,^22^,^23^,^24^,^25^,^26^,^27^,^28^,^29^,^30^,^31^,^32^,^33^. Contact-separation mechanism based TENGs use triboelectric effect to generate surface charges from periodic contact and separation between triboelectric layers. These surface charges are then utilized as a variable capacitor system by changing the gap between two triboelectric layers, to convert mechanical energy into electrical energy. Apart from material selection and device structure, another crucial factor affecting the performance of contact electrification process is surface topography of triboelectric contact surfaces. It plays a pivotal role in the charge generation process during contact electrification^34^,^35^,^36^. To improve the surface topography and properties, different methods have been used including soft lithography^21^,^37^,^38^, plasma etching^22^,^39^,^40^,^41^, nanoparticle deposition^42^ block copolymer self-assembly^43^,^44^ and chemical treatment^45^,^46^,^47^. The aforementioned fabrication methods and techniques are high cost, non-scalable, and can only be used for fabricating relatively small sized samples. Triboelectric nanogenerators hold huge potential for commercial applications in the area of energy harvesting and sensing devices. However, scalability and cost of fabrication techniques still remains a critical issue for mass production of triboelectric mechanism based devices on a commercial scale. Moreover, scalability of fabrication processes is also important in order to realize large scale nanogenerators, which can be used to harvest mechanical energy from sources such as vehicles, human walking and ocean waves.

Current TENG fabrication processes are limited by the wafer or chamber size of fabrication setup which in turn limits their suitability to only small sized samples. In this paper, we demonstrate a process flow for fabrication of triboelectric nanogenerators by fabricating components using large scale processes with high throughput. Roll-to-roll UV embossing^48^ was used to pattern large size polymer films without any restriction on the length of the film fabricated. We used lamination technique to fabricate large scale copper film on top of liquid crystal polymer (LCP) to be used as an electrode and triboelectric layer. Commercially available indium tin oxide (ITO) on large size polyethylene terephthalate (PET) was also tested as a triboelectric layer for LS-TENG. These components are then integrated together to realize large scale triboelectric nanogenerator (LS-TENG). These LS-TENGs hold great potential in harvesting mechanical energy on a larger scale e.g. from vehicle motion and human motion by embedding them in roads, pathways and indoor floorings. The fabricated LS-TENG generated a power output of 62.5 mW m^−2^ using palm tapping at 4 Hz. Current device use patterned PET as a triboelectric layer due to roll-to-roll process limitations, the device performance can further be improved by adapting the process for more efficient triboelectric materials and coatings such as polytetrafluoroethylene (PTFE).

Furthermore, roll-to-roll process based fabrication has also been proposed and demonstrated for fabricating large size pressure (force per unit area) sensor arrays. Triboelectric mechanism based sensor arrays have earlier been demonstrated for self-powered tracking systems^49^, tactile imaging^50^ and displacement sensor systems^51^. These sensor arrays were fabricated either by assembly of individual triboelectric devices in array form or use of wafer level fabrication process for patterning pixels array on polymer films. In this paper, we demonstrate a large size sensor array using large scale polymer films fabricated through scalable fabrication processes. The fabricated device is characterized as a self-powered pressure sensor array with a detection sensitivity of 1.33 V kPa^−1^. We conducted experiments to demonstrate practical applications of these sensor arrays for self-powered motion tracking and posture monitoring. The proposed roll-to-roll process based fabrication steps are suitable for implementing for industrial scale fabrication to achieve economies of scale. These processes enable extreme scalability at low cost, and thereby making it highly attractive for wide range of applications such as motion tracking, sports/athletic training, electronic skin and remote patient monitoring.

## Results

### Fabrication

**Figure 1:**
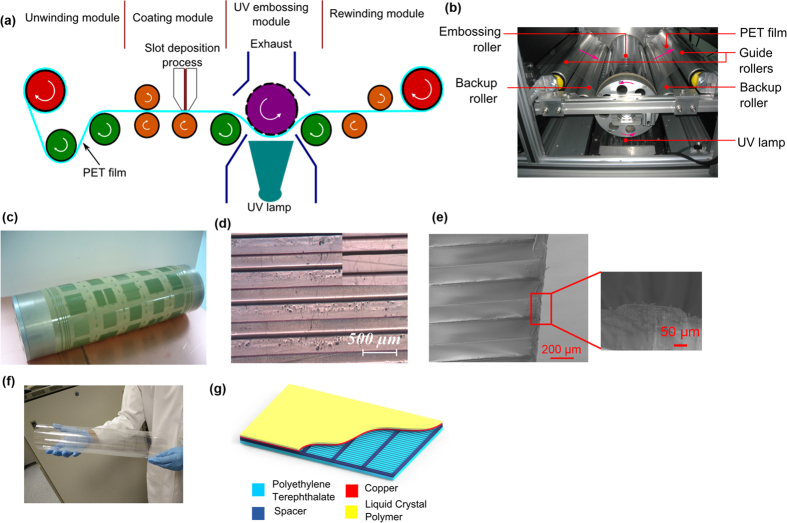
(**a**) Schematic of roll-to-roll UV embossing fabrication setup. (**b**) Photograph of the roll-to-roll fabrication setup. (**c**) A mold made of polyurethane based photopolymer attached in embossing roller. (**d**) Optical image of line patterned film. (**e**) SEM image of the line patterned film. Inset shows the SEM image of the cross section. (**f**) Large patterned PET sheet fabricated for triboelectric energy harvesting applications. (**g**) Schematic of LS-TENG.

For LS-TENG, patterned PET film and copper film are used as two triboelectric layers due to their relative tendency to attract and donate electrons, respectively. For the fabrication of patterned PET triboelectric layers, roll-to-roll UV embossing^48^ is used. The setup for roll-to-roll fabrication process is shown in Fig. 1a,b. The UV embossing system consists of four modules that are: (1) unwinding module for supplying substrate film that is a blank PET film; (2) coating module for depositing UV curable resin on PET film; (3) UV embossing module for patterning microstructures on PET film and (4) rewinding module that provides web tension for separating the embossed PET film from the embossing roller and then rewinds for collecting the embossed PET film. UV curable resin is coated on PET films using a slot die in the coating module. The coating thickness of 20~60 μm with thickness uniformity better than +/− 10% can be obtained for the layer. Large area patterned PET film is fabricated via roll-to-roll UV embossing inside UV embossing module with UV exposure. Figure 1b,c show images of the fabrication setup and embossing roller, respectively. Optical and SEM images of a patterned PET film with line patterns (line pitch: 500 μm) is shown in Fig. 1d,e, respectively. A photograph of fabricated patterned PET film sample is shown in Fig. 1f. Large patterned films were cut into size of 40 cm × 40 cm size sheets. These patterned PET sheets are used to increase the performance of triboelectric effect based contact electrification^34^. The patterned PET film fabricated using roll-to-roll UV embossing acts as one of the triboelectric layer in the large scale LS-TENG. For the second triboelectric layer, an 18 μm thick copper film attached on top of 50 μm thick LCP substrate is used. This layer is fabricated by laminating the copper film on LCP film. Thereafter, 1 mm thick foam tape is used as spacer and assembled on top of patterned PET film. The copper film is then assembled on top of the foam tape spacer (see Supplementary Fig. S1). Due to the large size of patterned PET film, the film tends to sag down permanently onto the copper electrode which hinders the contact separation mechanism required for charge generation for mechanical energy harvesting. Therefore it is important to assemble spacers at regular spacing between copper and patterned PET triboelectric layer to help the large patterned polymer film rebound back to its original position after it is subjected to mechanical force. A schematic diagram of LS-TENG is shown in Fig. 1g. Apart from Cu film on LCP, commercially available PET coated with ITO film was also used and tested as second triboelectric layer and electrode integrated with patterned PET film to fabricate LS-TENG.

### Effect of different embossed patterns

**Figure 2:**
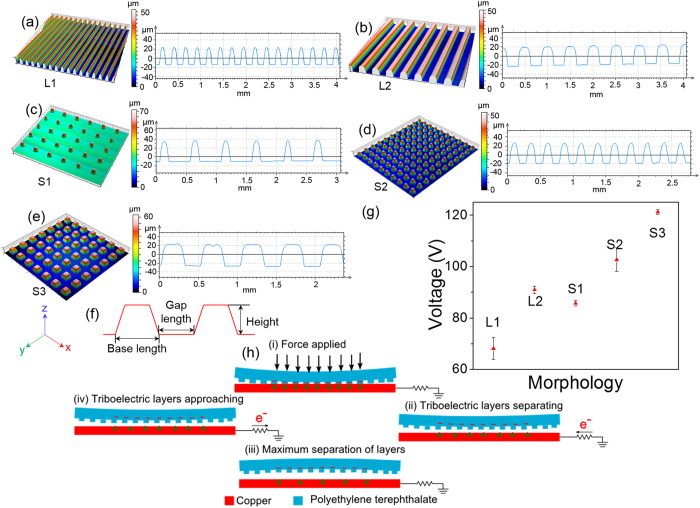
(**a–e**) 3-D morphology and cross section of different embossed patterns. (**f**) Geometrical parameters of different embossed patterns. (**g**) Voltage output generated by the different patterns under same force level.

To study the effect of embossed patterns on the performance of triboelectric mechanism, different samples were fabricated. We fabricated two types of line patterns and three types of square shaped patterns. The topography of these five patterns are shown in Fig. 2a–e. The geometric parameters for the line and square patterns are given in Table 1. As shown in the cross section profiles in Fig. 2a–e, side walls of embossed structures are not perfectly vertical due to fabrication process limitation. Also, the top of the embossed structure was observed to be curved instead of completely flat. This results in reduction of effective area at the top of embossed structures as compared to the base area of the embossed structures. At the same time, embossing of structures is important as microstructured films results into improved performance of the triboelectric performance. This is due to easier charge separation and enhanced triboelectric effect exhibited by the patterned films as demonstrated by Fan *et al.*^36^. These two effects work in contrast with each other affecting the performance of TENG. The samples were tested using a mechanical stage to examine the performance of different morphologies. The order of the performance of different morphologies was observed as under:


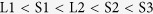


Amongst L1 and L2, the designed base area of the structures was same but because of tapered shape of fabricated structures due to process limitation, the effective contact area at the top was reduced for L1. This resulted in better performance of L2 compared to L1 in spite of same base area of embossed structures in both the cases. Amongst S1, S2 and S3, S1 was designed to have minimum base area and was observed to have the least performance. S2 and S3 were designed to have same base area but due to fabrication process limitation, the S2 had a lesser top contact area as compared to S3. It was observed that S3 had a significantly better performance compared to S2 which is in accordance with the results observed for L1 and L2.

### Working mechanism

The schematic for operating mechanism of LS-TENG is shown in Fig. 2h. Initially, both the triboelectric layers, i.e. patterned PET film and copper film are uncharged. During the device operation, the copper film was kept at bottom while patterned PET film is at the top separated by spacers from copper film. As there is any mechanical force applied on the PET film due to activities like walking, palm tapping etc., the electrons are injected from the copper film into patterned PET layer owing to copper’s higher tendency to donate electrons. The charges on the patterned PET film are preserved due to its insulating properties, whereas the charges on conductive copper film can flow through the load resistor connected to the ground as electric potential changes at the copper electrode. After the applied force is released, patterned PET film start separating from the copper film. This results into an increase in the potential at copper electrode, leading to the flow of electrons from the reference ground electrode towards copper electrode. As the patterned PET film separates due to the stored elastic energy stored when applied with a mechanical force, it reaches a maximum point of separation between the two triboelectric layers. At the maximum separation point, there is no flow of electrons in the load resistor connected between electrode and ground. Thereafter, the patterned PET film starts approaching towards the copper film leading to flow of electrons in the load resistor in opposite direction. This is equivalent of the one cycle of electricity generation due to application of force on LS-TENG.

### LS-TENG performance and applications

**Figure 3:**
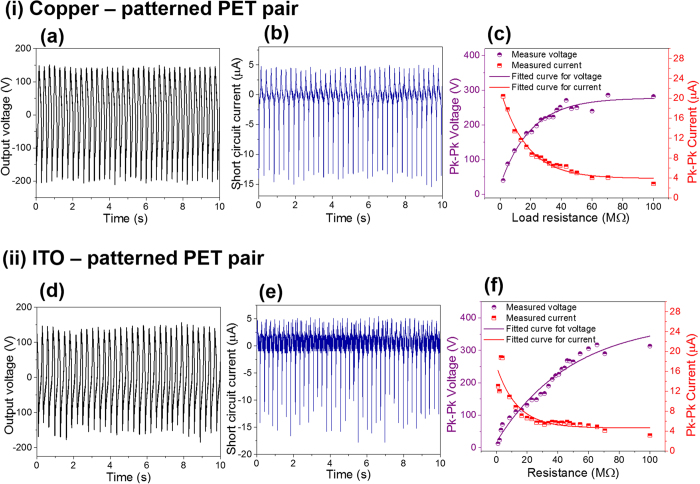
(**a**) Output voltage and (**b**) current generated by using LS-TENG using copper as second triboelectric layer. (**c**) Dependence of peak-to-peak (Pk-Pk) voltage and current on load resistance. (**d**) Output voltage and (**e**) current generated by using LS-TENG using ITO as second triboelectric layer. (**f**) Dependence of peak-to-peak (Pk-Pk) voltage and current on load resistance.

From the application perspective, it is important that the energy harvesting devices be characterized in practical scenarios. The energy harvesting characteristics were measured using palm tapping on LS-TENG using line pattern L2 as a triboelectric layer. The peak-to-peak output voltage was measured to be 344.63 ± 1.37 V when tapped using palm (Fig. 3a) using copper and patterned PET film as a triboelectric pair. The peak-to-peak short circuit current generated by LS-TENG was measured to be 18.12 ± 0.13 μA as shown in Fig. 3b. To study the variation of power transferred to a load resistor, LS-TENG was connected to different load resistors. The peak voltage measured across the load resistor connected to LS-TENG increases continuously as the value of load resistance increases, as shown in Fig. 3c. On the other hand, the current flowing across resistor decreases as the value of load resistor increases. The peak power density using the active area used for energy generation, was measured to be 62.5 mW m^−2^. Using ITO and patterned PET as a triboelectric pair, the peak-to-peak output voltage and current generated were measured to be 315.10 ± 16.69 V and 17.66 ± 0.33 μA. The peak power density from the device was measured to be 53.82 mW m^−2^. The optimum performance of Cu based LS-TENG was observed to be slightly better than ITO based LS-TENG device. It has to be noted that the current output of the device is still low in spite of large area of the device. The low current output of the device can be attributed to the limited utilization of the device area for triboelectric surface charge generation during the testing. The force was only applied to a limited area using palm tapping during testing to observe the energy harvesting characteristics of LS-TENG.

**Figure 4:**
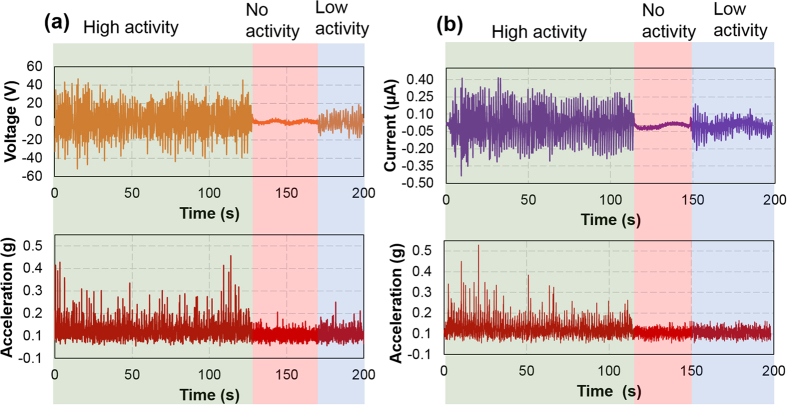
(**a**) Output voltage and (**b**) current generated by LS-TENG by natural movement of a human working on computer, when assembled at the back of an office chair. Corresponding acceleration measured was using an ADXL325 accelerometer.

The voltage and current output of Cu based LS-TENG were also tested at different frequency of applied force (see Supplementary Fig. S2). To demonstrate the practicality of LS-TENG, nearly 90 commercial LEDs were lighted up by directly connecting them to LS-TENG subjected to palm tapping (see Supplementary Fig. S3). To demonstrate the viability of LS-TENG in day-to-day applications, the device was assembled at the back of an office chair (see Supplementary Fig. S4). The output was recorded as the subject sat on chair and worked in three conditions: high activity, low activity and no activity. Here, the high activity and low activity refers to rocking of chair by the human subject and typing on the keyboard while working on a desktop by the human subject, respectively. These activities led to contact and separation between the triboelectric layers of LS-TENG, resulting in generation of output signals. In the no activity region, the human subject sat still on the chair and any signal produced were due to involuntary movement of the human subject. A commercial 3-axis accelerometer ADXL325 from Analog Devices, Inc. was assembled at the back of the chair to observe the activity levels. The total acceleration as experienced by the accelerometer assembled on the chair is shown in Fig. 4a,b. LS-TENG generated peak-to-peak voltage and current of up to 85 V and 0.75 μA, respectively in the high activity region. During low activity, LS-TENG produced peak-to-peak voltage and current of up to 35 V and 0.3 μA, respectively.

### Pressure sensor array

**Figure 5:**
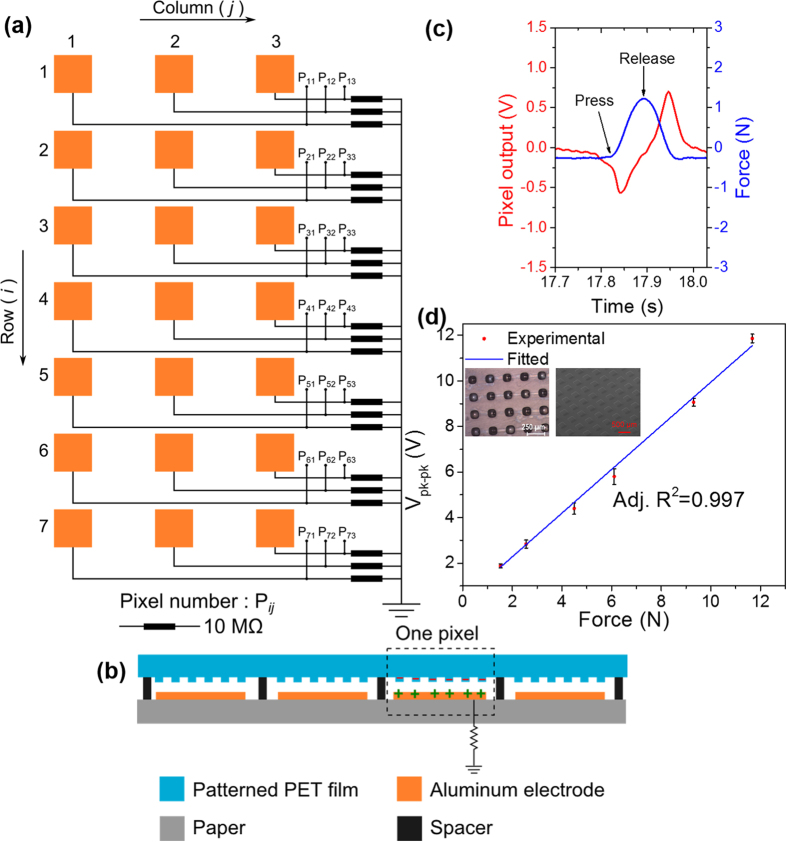
(**a**) Schematic diagram of the large scale triboelectric sensor array using roll-to-roll UV embossing process. (**b**) Configuration of the sensor array design. (**c**) Time domain signal generated by the pixel and the force sensor assembled beneath the pixel. (**d**) Peak-to-peak voltage generated by the pixel for different values of applied forces. Inset image shows optical and SEM images of the micropillar structures fabricated on PET sheet.

The fabrication process used for LS-TENG was also extended for fabricating low-cost pressure sensor arrays. For the fabrication of pressure sensor array, square patterns S3 as shown in Fig. 2e, were fabricated to form pixel patterns on the PET films using roll-to-roll UV embossing process as shown in the inset of Fig. 5d. For the electrodes of sensor array, we used commercially available stock paper as a substrate to make electrode patterns for individual pixels using aluminum foil as shown in Fig. 5b. 200 μm thick spacers were used to separate the aluminum electrode and individual PET patterns. The thickness of the spacer was reduced in order to reduce the value of minimum force required for contact and separation of both triboelectric layers to enable sensing of small values of force. Also, the spacers separating all the individual pixels helped in minimizing the cross talking between different pixels as external force is applied on the film. The basic sensing principle of the self-powered sensor array is similar to the energy generation mechanism as described earlier.

The fabricated sensor array can be used for motion tracking, tactile sensing, electronic skin, posture monitoring and various other applications. A schematic of 7 × 3 sensor array is shown in Fig. 5a. For every pixel, a load resistor of 10 MΩ is connected between the aluminum electrode and common ground. The pixels were tested using a commercial force sensor FSS1500NSB from Honeywell International, Inc. for calibration. The schematic for calibration setup is shown in Supplementary Fig. S5. As the pixel was stimulated using different levels of force, the signals were collected from both pixel output and the force sensor. Time domain signals generated by the pixel and force sensor are plotted on same time scale in Fig. 5c. As the pixel is pressed, initially there is a negative peak observed in the voltage signal generated across the load resistor as shown in Fig. 5c. As the force reaches a maximum value, two triboelectric layers are in contact and measured voltage across 10 MΩ resistor reaches zero. Thereafter, as the force is released, a positive peak is observed in the voltage signal generated across the 10 MΩ resistor. The peak-to-peak voltage values generated by the pixel are plotted for different values of force applied as shown in Fig. 5d. The peak-to-peak voltage was observed to increase linearly as the force level applied to the pixel increases. The force sensitivity of the sensing pixel was characterized to be 0.935 V N^−1^ which was equivalent to pressure detection sensitivity of 1.33 V kPa^−1^.

**Figure 6:**
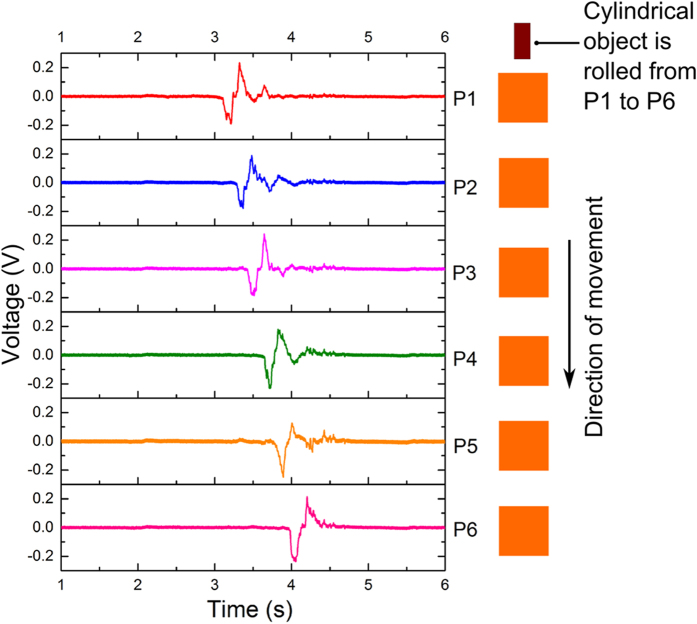
Time domain output voltage signals generated by an array of 6 pixels as a cylindrical object with a diameter of 9.2 cm, width of 1.9 cm and weight of 30 gm, is rolled from P1 to P6. The pixels generate a signal as the object contacts the pixel.

The sensor array was tested for motion tracking of a cylindrical object rolling over the array. A cylindrical object with a diameter of 9.2 cm, width of 1.9 cm and weight of 30 gm, was rolled on a linear array of six pixels from P1 to P6 as shown in Fig. 6. As the cylindrical object rolls over the sensors array, it leads to contact between patterned PET film and the aluminum electrodes. This in turn leads to a negative peak observed in the voltage signal generated by the pixel. As the object keeps rolling, a maximum peak force is reached leading to maximum contact between the patterned PET film and aluminum electrode. As the object leaves the pixel, triboelectric layers start separating from each other leading to a positive peak. This mechanism leads to a voltage signal generated, when the object rolls on top of the pixel. It is clear from the graph shown in Fig. 6 that the voltage pulse shifts to the right as object moves from pixel P1 to P6 and touches the pixels sequentially. The position data acquired using these time domain pulses can be utilized to calculate the velocity and acceleration of the object (see Supplementary Fig. S7 and Table S1). These low cost and large size sensor arrays can potentially be used for movement tracking of objects with applications in security systems, sports/athletic training and remote patient monitoring.

**Figure 7:**
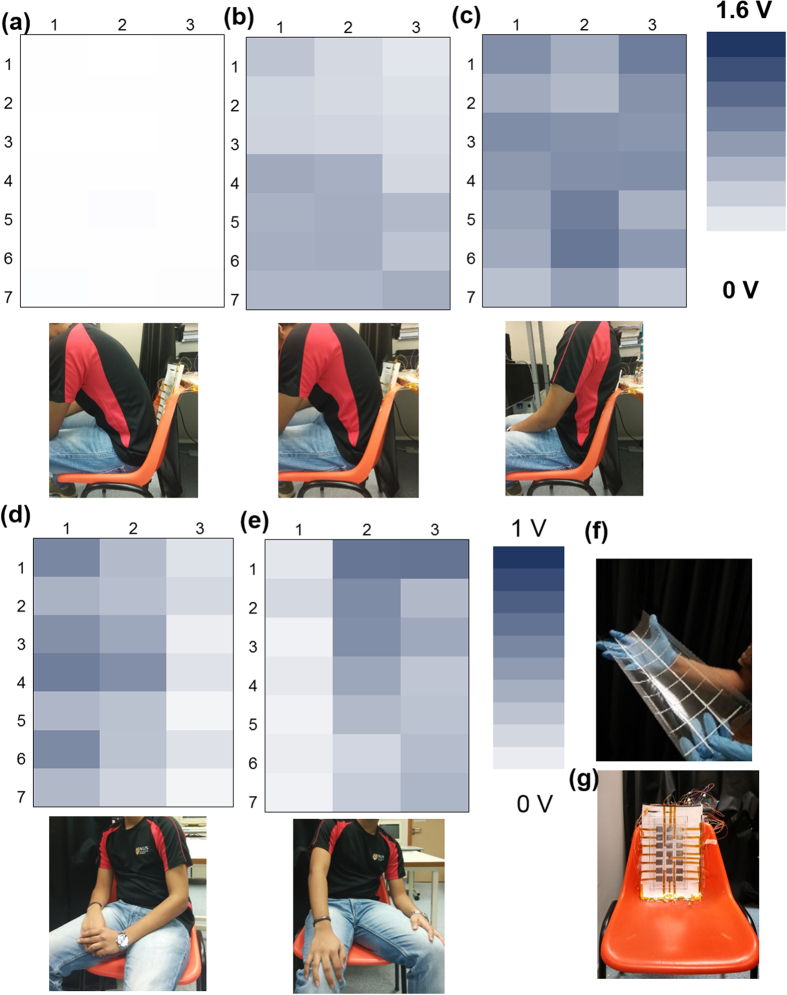
Heat map of a 7 × 3 sensor array for different posture when array assembled on back of chair. (**a**) Without touching the sensor array, (**b**) partially touching the sensor array pixels, (**c**) completely touching the sensor array pixels, (**d**) leaning on right side of the subject, (**e**) leaning on left side of the subject. (**f**) Photograph of patterned PET film fabricated using roll-to-roll fabrication process assembled with spacers. (**g**) Sensor array assembled on the back of a chair.

Thereafter, the sensor array was also demonstrated for application in posture monitoring. For experiments, the array was assembled at the back of a chair as shown in Fig. 7g. The sensor array pixels generate an electrical output as the subject touches the pixels of sensor array. The heat map of the peak-to-peak voltage generated by the pixels is shown in Fig. 7a–e for different positions of the subject. In position (a) the subject is not touching any pixels; as the subject moves to position (b), the subject contacts the majority of pixels in the lower half of sensor array (after row 3) that results in darker pixels in lower half as compared to the upper half. Also, it can be observed that there is some output observed in the pixels above row 3 as compared to the position (a). This can be attributed to cross-talking between pixels, as the patterned PET film deforms when applied with an external force. In position (c), the subject straightens the back and pressure is applied on whole array. Thereafter, Fig. 7d,e show the results as the subject tilts to right and left respectively (as seen from subject’s position). The experiments demonstrate that the sensor array can be used for applications like posture monitoring, sleep pattern monitoring, walking pattern monitoring and electronic skin applications.

In summary, we have demonstrated a process flow for large scale fabrication triboelectric nanogenerators with high throughput. Roll-to-roll UV embossing has been utilized to fabricate large size patterned polymer films to realize triboelectric nanogenerators. LS-TENG generated a peak-to-peak voltage and current of 344.63 ± 1.37 V and 18.12 ± 0.13 μA respectively. LS-TENG was demonstrated to generate a power of 62.5 mW m^−2^ using palm tapping. Roll-to-roll fabrication based process was also used to fabricate large size and low cost pressure sensor array with a detection sensitivity of 1.33 V kPa^−1^. We also conducted experiments using the sensor array for posture monitoring by assembling the array at the back of chair. The fabricated large size sensor array can be used in motion tracking, patient monitoring, posture monitoring and electronic skin applications. This work is aimed at developing and demonstrating scalable fabrication process for large scale production of triboelectric nanogenerators and sensor arrays with high throughput and low cost.

## Methods

### Fabrication of the Mold

Large area flexible molds for roll-to-roll UV embossing are fabricated via patterning a thin photosensitive polyurethane sheet by means of designing micro patterns, photolithography and development of the photosensitive polyurethane sheet in a chemical solution. The embossing roller is 50 cm long in axial direction with a diameter of 16 cm. The 1 mm thick patterned polyurethane sheet is then attached on the embossing roller by using double-sided adhesive tape. In order to obtain a roller mold with a seam as narrow as possible, the length in circumferential of the polyurethane flexible mold needs to cut precisely, taking into account of the thickness of double-side adhesive tape and thickness of the mold itself.

### Roll-to-Roll Embossing of Patterned PET Films

PET film is pre-coated with a primer layer, which consists of functionalized α-olefin containing copolymers and cross-linking agents, to improve the adhesion of coated UV sensitive resin layer during roll-to-roll UV embossing process. PET film with its thickness of 125 μm is first slot die-coated with a UV curable resin. The UV curable resin is a solvent-free acrylate-based lacquer with viscosity of about 100 centipoise at 25 °C. Thereafter the film is embossed using the embossing roller fabricated in previous step. The UV lamp is irradiated directly below the embossing roller using a UV lamp. It is used to cure the UV curable resin after the resin flows and fully fills the cavities on the mold. After the completion of coating, embossing and separation processes, micro patterns on the mold are transferred onto PET film. Hence, patterned PET film with micro patterns are obtained by means of roll-to-roll UV embossing.

### Electrical Measurements of LS-TENG

For voltage characteristics, LS-TENG was tested by connecting by using a 100 MΩ probe and connecting to a DSOX3034A oscilloscope. Current characteristics were measured by using a low noise SR570 current pre-amplifier. For measuring the power generation characteristics at different accelerations, commercial accelerometer ADXL325 was assembled at the back of the chair.

### Electrical Measurements of Sensor Array

For the sensor array measurement, load resistors of 10 MΩ were connected between the aluminum electrodes of 7 × 3 array and common ground. The output across these load resistors were measured using individual channels of USB-6363 data acquisition (DAQ) board from National Instruments. The output from 21 channels from USB-6363 was acquired using Lab View to observe the individual output from every pixel (see Supplementary Fig. S6).

### Calibration using force sensor

For calibration of sensor array, commercially available FSS1500NSB sensors from Honeywell International Inc. were fixed below the sensor array pixels. The sensors were biased using 5V direct current (DC) power supply (see Supplementary Fig. S5). The output from the pixel and force sensor were then measured in real time as the sensor pixel was stimulated using external force.

## Additional Information

**How to cite this article**: Dhakar, L. *et al.* Large Scale Triboelectric Nanogenerator and Self-Powered Pressure Sensor Array Using Low Cost Roll-to-Roll UV Embossing. *Sci. Rep.* **6**, 22253; doi: 10.1038/srep22253 (2016).

## Supplementary Material

Supplementary Information

## Figures and Tables

**Table 1:** Geometrical parameters for different fabricated morphologies.

	Pattern	Base length	Gap length	Height
Line patterns	L1	250	250	~45 um
	L2	500	500	~45 um
Square patterns	S1	300	700	~45 um
	S2	300	200	~45 um
	S3	600	400	~45 um
