# Supplementary material for: Large Scale Triboelectric Nanogenerator and Self-Powered Pressure Sensor Array Using Low Cost Roll-to-Roll UV Embossing
Source: Sci Rep. 2016 Feb 24;6:22253. doi: 10.1038/srep22253 (PMC4764913; doi:10.1038/srep22253)
Supplement: Supplementary Information [file srep22253-s1.doc]

Supplementary Information

Large Scale Triboelectric Nanogenerator and Self-Powered Pressure Sensor Array Using Low Cost Roll-to-Roll UV Embossing

Lokesh Dhakar, Sudeep Gudla, Xuechuan Shan, Zhiping Wang, Francis Eng Hock Tay, Chun-Huat Heng and Chengkuo Lee*

^1^ Department of Electrical and Computer Engineering,

National University of Singapore, 4 Engineering Drive 3, Singapore 117576

^2^ NUS Graduate School for Integrative Sciences and Engineering, Centre for Life Sciences (CeLS), 28 Medical Drive, Singapore 117456

^3^ Singapore Institute of Manufacturing Technology (SIMTech), 71 Nanyang Drive, Singapore 638075

^4^ Department of Mechanical Engineering, National University of Singapore, 9 Engineering Drive 1, Singapore 117576

*Corresponding Author: Chengkuo Lee

**E-mail:** [**elelc@nus.edu.sg**](mailto:elelc@nus.edu.sg)

Figure S1: Detailed schematic for the assembly of LS-TENG.

Note 1. Effect of frequency on the LS-TENG performance:

LS-TENG was tested at different tapping frequencies to observe the effect on device performance. Both the measured voltage and current were observed to increase as the tapping frequency was increased from 1 Hz to 5 Hz. The peak-to-peak voltage increased from 232 V to 486 V as the frequency increased from 1 Hz to 5 Hz. The peak-to-peak current increased from 7.2 µA to 21.2 µA as the frequency increased from 1 Hz to 5 Hz. The results are shown in Figure S3.


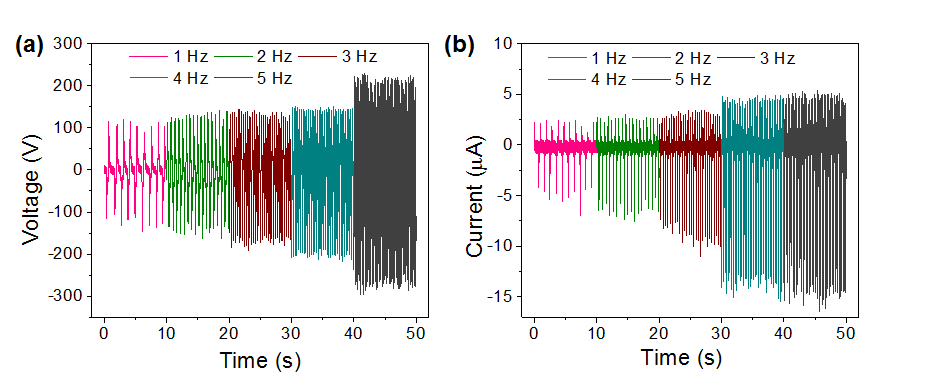


Figure S2: (a) Voltage generated by LS-TENG at different frequencies. (b) Current generated by LS-TENG at different frequencies.


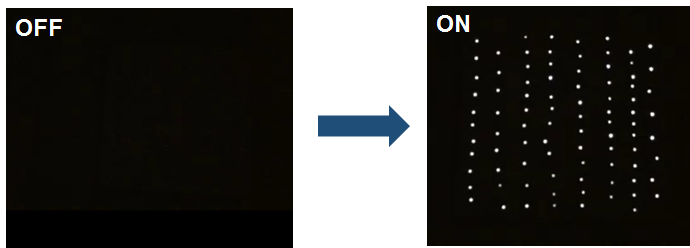


**Figure S3:** LEDs lighted up using hand tapping.


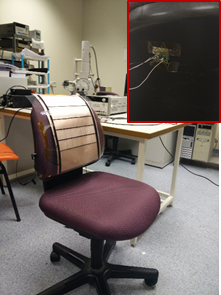


**Figure S4:** LS-TENG assembled on a chair for experiment. Inset: 3-axis accelerometer assemble at the back of chair.


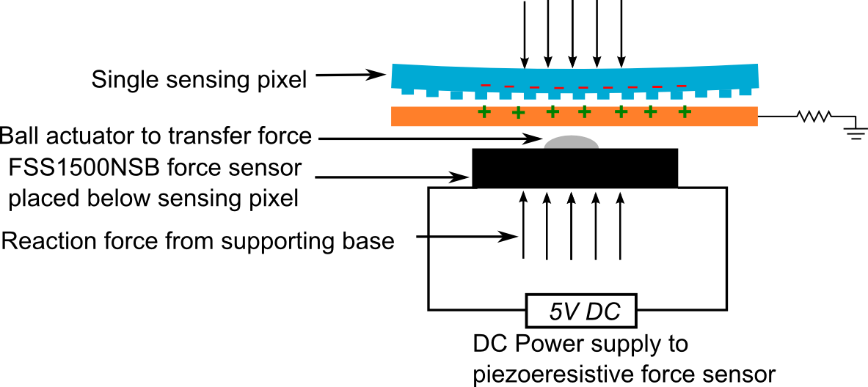


Figure S5: Sensor array output measurement setup.


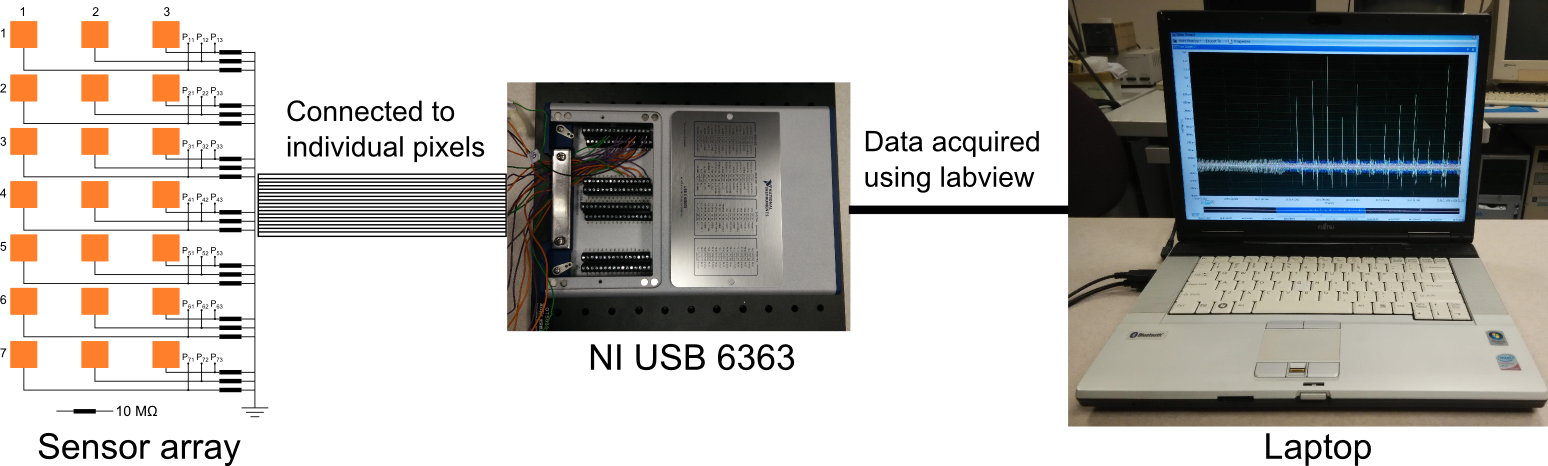


Figure S6: Sensor array output measurement setup.

Note 2. Calculation of average velocity and average acceleration using the position data:


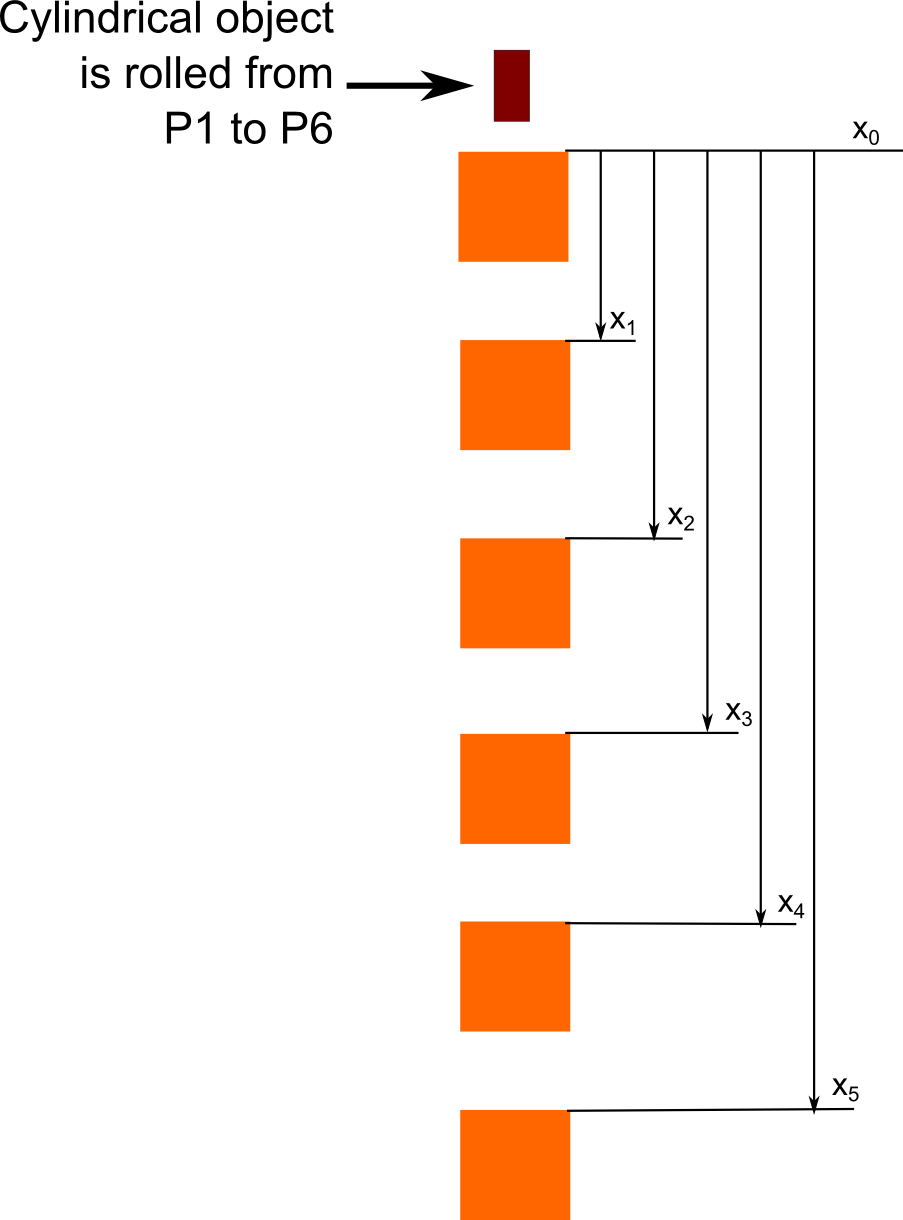


Figure S7: Labeling of the positions of the cylindrical object as it rolls from P1 to P6.

The position data can be acquired using the time domain signal from every pixel as shown in Figure 5.
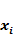
 is the displacement of the object from the initial starting position when it reaches the starting point of
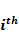
 pixel. This data can then be used to calculate the average velocity
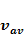
 using following equation:

| 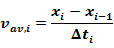 | (2) |
| --- | --- |

where,
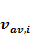
 is the average velocity of the object between
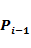
 and
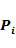
; and
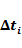
 is the time interval elapsed between
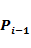
 and
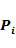
.

Similarly average acceleration
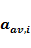
 can be calculated using the average velocity values calculated using Equation (2):

| 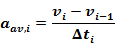 | (3) |
| --- | --- |

where,
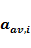
 is the average velocity of the object between
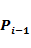
 and
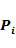
; and
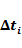
 is the time interval elapsed between
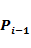
 and
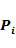
. Equations (2) and (3) were used to calculate the average velocity and acceleration values as shown in Table S1.

Table S1. Calculated values of average velocity and average acceleration

|  | Distance  [cm] | Time  [s] | Average velocity [cm s^-1^] | Average acceleration [cm s^-2^] |
| --- | --- | --- | --- | --- |
| x_0_ | 0 | 3.10 | 0 | - |
| x_1_ | 3.7 | 3.29 | 19.68 | 0.01 |
| x_2_ | 7.4 | 3.43 | 26.43 | 0.02 |
| x_3_ | 11.1 | 3.65 | 16.82 | -0.02 |
| x_4_ | 14.8 | 3.80 | 24.50 | 0.02 |
| x_5_ | 18.5 | 3.98 | 20.11 | -0.04 |
